# Predictive value of tissue oxygen saturation upon mortality in Emergency Department patients with sepsis

**DOI:** 10.1186/cc9461

**Published:** 2011-03-11

**Authors:** C Vorwerk, T Coats

**Affiliations:** 1 University Hospitals of Leicester, UK

## Introduction

Microvascular dysfunction and inadequate delivery of oxygen to the tissues is a feature of septic shock. The degree of this microcirculatory impairment has not been assessed in the early phases of Emergency Department (ED) sepsis management. The purpose of this study was to assess the relationship between tissue oxygen saturation (StO_2_) and conventional vital signs and in-hospital mortality for ED patients with severe sepsis or septic shock.

## Methods

Prospective cohort study of adult ED patients with severe sepsis or septic shock. Standard vital signs were monitored in all patients. StO_2 _measurements using near-infrared spectroscopy were commenced as soon as possible after the patients' arrival in the ED. The measurements were continued throughout the stay in the ED whilst receiving normal treatment. StO_2 _readings were repeated after 24 hours of sepsis management. All patients were followed up for 30 days.

## Results

Forty-nine patients were included in this study, of which 24 (49%) died. Nonsurvivors were significantly older than survivors (79 vs. 64, *P *= 0.008) but there were no significant differences in co-morbidities or conventional vital signs. On arrival in the ED there was no difference in mean StO_2 _between survivors and nonsurvivors (72% vs. 72%, *P *= 0.97). With treatment, StO_2 _improved significantly to 78% (*P *= 0.006) in survivors but remained persistently low in nonsurvivors. The AUROC for StO_2 _was 0.63 on ED departure and 0.71 after 24 hours of treatment, performing far better than heart rate (0.53), SpO_2 _(0.50) and systolic blood pressure (0.51). There was no correlation between StO_2 _and any of the routine vital signs.

## Conclusions

Our results demonstrate that a consistently low tissue oxygen saturation despite initial sepsis resuscitation is associated with an increased in-hospital mortality. We have further shown that tissue oxygen saturation is a better prognostic indicator than conventional vital signs in severely septic ED patients.

